# Similar Survival Rates of Territorial and Sneaker Males in a Polymorphic Damselfly: A Multi‐Year Study

**DOI:** 10.1002/ece3.72623

**Published:** 2025-12-10

**Authors:** Yoshitaka Tsubaki, Rowan E. Hooper, Stewart Plaistow, Yuka Samejima, Thomas N. Sherratt

**Affiliations:** ^1^ Center for Ecological Research Kyoto University Otsu Japan; ^2^ New Scientist London UK; ^3^ Institute of Infection, Veterinary & Ecological Sciences The University of Liverpool Liverpool UK; ^4^ Department of Biology Carleton University Ottawa Ontario Canada

**Keywords:** alternate reproductive tactics, Cormack–Jolly–Seber, damselfly, longevity, male polymorphism, mark‐recapture, survival

## Abstract

Male alternative reproductive tactics (ARTs) have been documented across diverse taxa and typically involve a male morph that defends resources to attract females and an alternate morph that exploits this behavior to obtain matings by stealth (territorial‐sneaker tactics). One might expect that territorial morphs would live shorter lives than sneaker morphs due to their higher vulnerability to predators, their propensity to get injured and higher energetic costs. To date, there have been few direct field tests of this prediction. A mark‐release‐resighting (MRR) study conducted in 1992 found that orange‐winged territorial morphs of the damselfly *Mnais costalis* had on average a shorter minimum reproductive lifespan than clear‐winged sneaker morphs, if one assumed that they all emerged on a single day, but not if they were assumed to be of age 0 at the time of capture. We fitted Cormack–Jolly–Seber models to these 1992 data, as well as MRR studies conducted on *Mnais costalis* in four subsequent years. These models allowed us to jointly estimate the daily survival and resighting rates of the morphs and identify consistent predictors of their survival and detectability. There was no evidence of a significant difference in survival between male morphs or between males and females, although daily survival did consistently rise then fall with time since capture (age) and consistently declined with temperature above 17°C. By contrast, there was strong evidence that the resighting rates of individuals differed among morphs, with orange‐winged males being more detectable. While there is no compelling evidence that the territorial and sneaker morphs have different daily survival rates, this does not preclude the possibility that orange‐winged territorial males have high reproductive success on the days they are able to maintain a territory while the reproductive success of clear‐winged sneaker males has reproductive success that is more broadly distributed across their lives.

## Graphical abstract

Male *Mnais* damselflies occur in two genetically controlled morphs: an orange‐winged territorial morph that defends oviposition sites and actively courts females and a co‐occurring clear‐winged sneaker morph that does not defend territories or display, yet mates opportunistically with females. One might expect that territorial morphs would live shorter lives than sneaker morphs because of their greater energetic costs and higher risk of injury, and an earlier analysis indicated that this was indeed the case. Here, we analyze five field seasons of mark‐recapture data going back three decades using modern methods and find there is no evidence that the two male morphs differ in their survival rates.

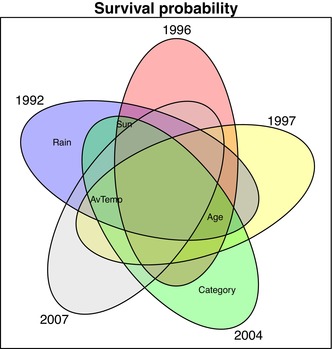

## 1 Introduction

Many animal species have two or more distinct male forms, each exhibiting a different reproductive strategy. These “alternative reproductive tactics” (ARTs) have been documented across diverse taxa (Gross 1996; Shuster and Wade 2003; Taborsky et al. 2008; Neff and Svensson 2013). Although details vary, male ARTs typically follow “producer‐scrounger” (“territorial‐sneaker”) roles, with “bourgeois” males defending territories or nests in which females will reproduce, and “parasitic” males exploiting the reproductive resource maintained by their conspecifics (Engqvist and Taborsky 2016). In the ruff ( *Philomachus pugnax* ), for instance, “independent” males establish and defend mating territories, actively courting females, whereas “satellite” males occupy territory edges and attempt to sneak copulations when the territory holder is distracted (Widemo 1998). A similar set of strategies appears in salmonid fishes where large, dominant “hooknose” males monopolize spawning sites and court females, while smaller “jack” males lurk nearby, attempting to fertilize eggs once spawning has occurred (Tanaka et al. 2009).

In many species that exhibit ARTs, the strategy adopted by individuals is conditional on its developmental environment, and in some cases may even be flexible. For example, developmentally arrested adult male orangutans, which can act as sneakers, tend to remain arrested if there are mature males in the group, but can subsequently develop into mature males with flanges (Kunz et al. 2023). By contrast, in species like the ruff, independent and satellite males are genetically determined (Loveland et al. 2025). To understand how such genetic polymorphisms evolve and persist, researchers have typically invoked frequency‐dependent selection in which strategies are favored when rare, but disfavored when common (McNamara and Leimar 2020; Broom and Rychtář 2022). Here, the average long‐term fitness of the two tactics will necessarily be equal at equilibrium. In support, a recent meta‐analysis revealed that there was no strong evidence of a difference in reproductive success between male ARTs when the tactics were fixed at birth (Da Silva et al. 2025).

Despite the widespread belief that males with fixed ARTs have equal long‐term reproductive success, territorial males typically have conspicuous signaling traits, and actively seek to attract females, which may make them more vulnerable to predators (Godin 1995; Toivanen et al. 2009; Kuchta and Svensson 2014). Territorial males may also have to work harder to maintain territories than nonterritorial males and fight with conspecifics to retain possession (Vande Velde and Van Dyck 2013; Ord 2021). One might therefore wonder whether territorial‐sneaker ARTs typically involve a trade‐off between reproduction and longevity (Widemo 1998; Plaistow and Tsubaki 2000; Bretman et al. 2013), with territorials “living fast and dying young” and sneakers “living slow and dying old”. Widemo (1998) for example noted that the male territorial ruff did not eat or drink while on the lek and conjectured that territorial males may live shorter lives than satellite males due to their higher energetic stress. This general prediction has been rarely tested, although it has been addressed in some shorter‐lived organisms with male ARTs, notably damselflies (Odonata: Zygoptera).

The damselfly *Paraphlebia zoe* (Thaumatoneuridae) has two discrete male morphs: a territorial, aggressive form of male with colored wings (a black spot and a white stripe, BW) and a less territorial, less aggressive form with clear (hyaline) wings (HW, Rivas‐Torres et al. 2019). The male tactics appear to be genetically determined and they broadly follow territorial and sneaker roles (Rivas‐Torres et al. 2019). Despite these different tactics, MunguÍa‐Steyer et al. (2010) conducted a mark‐recapture experiment and found no evidence of a difference in daily survival between BW and HW males, although they did find that BW males had significantly higher daily resighting rates than HW males.

Perhaps the best‐known examples of genetically determined male mating strategies in damselflies are from the genus *Mnais* (Calopterygidae). The adult males of *Mnais costalis*, occur as one of two morphs characterized by wing color (Tsubaki 2003). Orange‐winged males tend to be territorial, defending prime oviposition sites by chasing away any intruders. By contrast, clear‐winged males tend to behave as nonterritorial “sneakers,” perching inconspicuously around occupied territories (Nomakuchi et al. 1984). Encounters between orange‐winged residents and neighboring territory holders or orange‐winged males that have not secured a territory (“floaters”, Watanabe and Taguchi (1990)) can escalate into aggressive skirmishes. Sneakers, too, may fight for a better vantage or a chance at mating, but clear‐winged males typically retreat when chased by an orange‐winged territorial (Nomakuchi et al. 1984; Hooper et al. 2006).

Tests of whether orange and clear‐winged male *Mnais* damselflies differ in their daily rates of survival have so far led to mixed results. Nomakuchi et al. (1988) analyzed mark‐recapture data to compare the survival rates of 37 marked orange‐winged and 16 marked clear‐winged male *Mnais pruinosa* and found no significant difference in the mean survival rates of the two male forms, based on comparing the slopes of the regression lines on their log survival curves. However, the low sample sizes suggest that the test was of limited power. Tsubaki et al. (1997) reported a more comprehensive mark‐recapture experiment involving over 200 marked individuals of each male morph that were seen again after the first capture. Using the time difference between the day of last sighting and the day of first capture as an indicator of longevity, they found no evidence of a difference in the survival distributions of the two male types. The authors did however find that clear‐winged males tended to live longer if one was prepared to assume that all the marked individuals emerged on the same day (1st June). As the authors note, using the time between last resighting and initial marking as a surrogate of longevity can have limitations, not least because it does not consider any differences in the probability that the two male morphs are resighted. Our Supporting Information (see also Figure S1) demonstrates that despite this reservation, the cumulative survival curves based on minimum lifespans can provide a good approximation of true survival. However as anticipated, a bias is introduced if the study ends while there are still marked individuals alive in the population, with true survival being more likely to be underestimated in individuals that are hard to detect. Overall, since clear‐winged males may be less conspicuous than orange‐winged males in appearance and behavior, one might expect them to be more subject to this bias. Indeed in one of the first mark‐recapture studies to estimate the density and survival rates of *Mnais pruinosa*, clear‐winged males were excluded from analysis because of their lower resighting rates (Higashi 1976).

In their review of mark‐recapture experiments in odonates, Sanmartín‐Villar and Cordero‐Rivera (2022) highlighted the need to study differences in the survival rates between male morphs of polymorphic species, and this is precisely what we have done here. Thus, we present a detailed analysis of five separate mark‐recapture studies (1992, 1996, 1997, 2004, and 2007) involving over 3800 marked *Mnais*, conducted by lead author Y.T. and colleagues over the past 30 years. In each case we fit Cormack–Jolly–Seber (CJS) models (Lebreton et al. 1992) which distinguish between the daily probability of an individual surviving between successive days (ϕ) and the probability of it being resighted on any given day (*p*). Separate estimation of these parameters is possible because if an individual is seen on Day 1 and seen again on Day 4 then we know it has survived so we can estimate its resighting probability from days it was seen and not seen. Similarly, if an individual is marked on Day 5 and is not seen again despite a high estimated probability of resighting, we can infer that it has most likely died after sufficient days have passed. We can model variation in both *ϕ* and *p* by making them dependent on environmental variables (daily sunshine, rain and mean temperature) as well as biological variables (body size, sex, morph if male, and age) and variables that might reflect a combination of environmental and biological variables (time in season).

The 1992–2004 data sets focused on *Mnais costalis*. However, the 2007 data set was a mark‐recapture study on sympatric *Mnais costalis* and *M. pruinosa*. Where these closely related species occur alone, they exhibit the same male‐limited color polymorphisms (Tsubaki and Okuyama 2016; Futahashi 2017). However, consistent with the character displacement that can arise when two polymorphic species occur together (Pfennig et al. 2006; Pfennig and Pfennig 2009; Tsubaki and Okuyama 2016; Sherratt et al. 2025), where their ranges overlap in central Japan, the *M. costalis* males in many populations (including the 2007 study site) are uniformly orange‐winged and the *M. pruinosa* males uniformly clear‐winged (Tsubaki and Okuyama 2016). There were therefore four different categories of marked adult in this population (female *pruinosa*, clear‐winged male *pruinosa*, female *costalis*, and orange‐winged male *costalis*), offering an opportunity to compare not just survival rates between morphs but also species.

Our primary aim was to formally evaluate the strength of evidence that the two male morphs of *M. costalis* differ in their daily survival once we account for differences in resighting rates. In addition, we wished to contribute to our understanding of odonate biology in general, by elucidating how a range of environmental and individual factors influence both their survival and resighting rates. Somewhat surprisingly, despite the increased statistical power and biological insights that one can gain by including environmental covariates such as rainfall and temperature as predictors of survival and resighting, they have been included in relatively few (~14%) of the recent mark‐recapture studies on odonates (Sanmartín‐Villar and Cordero‐Rivera 2022).

## 2 Materials and Methods

The study locations, and number of each sex, morph and age class of *Mnais* marked are summarized in Table 1, along with the start date and duration of the study season. The 1992 data have previously been presented (Tsubaki et al. 1997) but this study focused on comparing estimates of the minimum longevity (defined as the time difference in days between last and first sighting) of male *M. costalis* morphs. Similarly the 2007 data have previously been presented (Tsubaki and Samejima 2016) but this study sought to elucidate the relationship between the minimum reproductive lifespan of a male and the openness of the territory it defended. The other 3 years of data have not been analyzed. This paper therefore presents the first fit of CJS models to any of these data sets and the first to directly control for resighting rates. Individuals that were not measured for size (length of hind wing) were not included in our analysis of the first four data sets, so slightly more individuals were marked in these studies than indicated in Table 1. Since only about 60% of individuals were measured in 2007, we did not fit models with size as a predictor for that year. The study designs in each year involved noting the location of marked individuals along the stream when first seen on any given day. Their movement was not part of our primary analysis, but we return to the implications of local dispersal in our discussion.

**TABLE 1:** The site locations, start dates, study duration, number of visits and demographic details of the five mark‐recapture studies conducted on *Mnais costalis* in Japan.

Male morph/sex	Gozenyama, Ibaraki prefecture, 15th May 1992		Gozenyama, Ibaraki prefecture, 20th May 1996		Gozenyama, Ibaraki prefecture, 12th May 1997		Gozenyama, Ibaraki prefecture, 8th May 2004		Otsu, Shiga prefecture, 7th May 2007	
	71 days, 43 visits		64 days, 30 visits		47 days, 23 visits		63 days, 39 visits		45 days, 20 visits	
	Teneral	Mature	Teneral	Mature	Teneral	Mature	Teneral	Mature	Teneral	Mature
Orange	127	165	52	224	16	128	110	224	5 *costalis*	189 *costalis*
Clear	72	244	10	229	10	106	43	188	8 *pruinosa*	234 *pruinosa*
Female	95	325	13	310	6	128	80	282	4 *costalis*/10 *pruinosa*	110 *costalis*/99 *pruinosa*

*Note:* In Gozenyama *M. costalis* does not co‐occur with any other *Mnais* species and its males are polymorphic. In Otsu (the location of the 2007 study) *M. costalis* co‐occurs with *M. pruinosa* . Here, *M. costalis* males are monomorphic orange‐winged, whereas *M. pruinosa* males are monomorphic clear‐winged. Here, teneral refers to any individual considered pre‐reproductive at the time of capture.

### 2.1 Study Site Information

#### 2.1.1 1992, 1996, 1997 and 2004 Data

The study site was a narrow mountain stream in Gozenyama, Ibaraki prefecture, eastern Japan (36°33′N, 140°17′ E; elevation, 50–150 m). The study population was delimited at the downstream part of the site by a large weir, and by the altitudinal limit of the population 250 m upstream. Vinyl tags were placed every 2.5 m along the stream to facilitate recording the location of individuals. Meteorological data were obtained from the Mito meteorological observatory (36°22.8′N, 140°28′ E; elevation 29 m).

#### 2.1.2 2007 Data

Field censuses and behavioral observations were conducted in a riparian habitat surrounded by bushes and trees in Otsu, Shiga, Japan (34° 57′ N, 135° 56′ E; elevation 100–300 m). A 500‐m stretch of the stream was used for this study. Vinyl tags were placed every 5 m along the stream to facilitate recording the location of individuals. Meteorological data were obtained from the Shigaraki meteorological observatory (3455° 55′ N, 136° 50′ E; elevation 265 m).

### 2.2 Marking Procedure

All captured adults were individually marked on their hind wings with enamel pens. The size of the individual's left hind wing and its abdomen length were measured at the time of capture with a ruler accurate to 0.5 mm. Most adults emerged from streams over a short period (about a week). Newly emerged (teneral) individuals could be readily identified since they have a soft, shiny cuticle for 2 days after emergence (Corbet 1999). Due to their delicate nature, researchers did not mark freshly emerged individuals, but they were able to mark any later‐stage tenerals encountered if their wings had sufficiently hardened. Another diagnostic feature used to help age captured males was the color of their pterostigma. In *Mnais* damselflies several wing cells, found at the intersection of the distal margin of the costa and radius, are pigmented. Known as the pterostigma, the pigment is white in all pre‐reproductive individuals, but in reproductively mature males of both types, the pterostigma turns red (Watanabe and Taguchi 2000). In females it remains white throughout their life. The rigidity of the cuticle, the color of the pterostigma and the reflection of wings and body therefore enabled us to class individuals as pre‐reproductive or mature at the time of capture. After about a week of feeding and developing their flight muscles, adult *Mnais* damselflies tend to commence reproductive behavior (Watanabe and Taguchi 2000).

### 2.3 Statistical Methods

To estimate time‐ and age‐dependent survival rates of male and female *Mnais* damselflies while controlling for their resighting probabilities, we employed the package *marked* (Laake et al. 2013) from the R programming environment (R Development Core Team 2024). The program *marked* shares many features with the program *RMark* (Laake 2013) which in turn was developed to help construct models for the comprehensive mark‐recapture analysis software MARK (White and Burnham 1999). Originally developed to improve on execution times, the fact that *marked* is a stand‐alone open‐source package renders it attractive for generating reproducible research.

As with many programs analyzing mark‐recapture data, the program *marked* considers the mark‐resighting histories of individuals, with encounters in an observation period (here, a day) coded as 1 (starting with the first day it was caught and marked) and the absence of encounter coded as 0. Resighting rates and survival are jointly modeled, with the understanding that the survival estimates represent the joint probability of surviving and not emigrating from the study area between searches (“apparent survival,” Lebreton et al. 1992). Individual‐based predictors of the mark‐recapture sequence such as an individual's category (orange‐winged, clear‐winged, female) and its hind wing length were entered as additional columns in the data frame with a single value of each predictor for each individual's capture history. Time‐varying predictors which were the same for all individuals but varied by day (mean temperature, rainfall and sunshine per day) were coded as separate columns in the data frame, with each column referring to the base name of the predictor and the day in question (e.g., rain23, rain24, rain25). For CJS models, the default fields in the *marked* package that do not need to be directly coded include the age of the individual (considered as the time since it was first encountered and marked, unless the variable initial.age is specified in the input data) and the time in the experimental period (the same for all individuals, with time 0 representing the time the very first individual was captured). These parameters can be treated as factors (by referring to the variables in lower case, i.e., age and time) or as numerical covariates (by referring to the variables in upper case, i.e., Age and Time). Rather than constrain Age (coded as a covariate), hindwing size, average temperature, rainfall, and sunshine to be linearly related in a monotonically increasing or decreasing fashion to the log odds of resighting or recapture (see below), we allowed these predictors to deviate from logit linearity by fitting them as part of a polynomial spline using the splines2 package (Wang and Yan 2021)—see Laake et al. (2013) for a worked example. When evaluated, models with these splines generally had lower AIC than identical linear models without these splines, indicating that the additional parameters allowing further curvature were justified.

Naturally, the probability of observing a particular capture history depends on the underlying survival and resighting rates. Variation in survival and resighting rates can in turn be modeled through a combination of static (e.g., sex, morph, size) and time‐varying (e.g., age, temperature, rainfall) predictors that are combined in given ways using specified CJS model formulae (see below). In all cases a logit link function was assumed, ensuring that resighting and survival probabilities were between 0 and 1. Parameter values in each CJS model were estimated through maximum likelihood.

The details of model fitting using *marked* were as follows. Prior to fitting models, a *process.data* function was used to account for attributes specific to the data being analyzed, notably the time intervals between capture occasions (since sites were not visited every day, see Table 1). A *make.design.data* function, which combines the lists generated by *process.data* with the lists of model parameters (*design.parameters*) was then used to create the model structure as it relates to the data. Specific models were fitted to the data with the capture–recapture model (*crm*) function, which use both the processed data list and design data list as arguments, but also the formulae as to how the daily survival probability ϕ and resighting probability *p* varies with predictors such as sex, morph, size, and time in season. The models with the lowest out‐of‐sample deviance were identified using Akaike Information Criteria (= −2 log likelihood +2 number of parameters).

### 2.4 Model Comparison

Since our primary aim was to elucidate the strength of differences in the daily survival of the different male morphs and sexes while controlling for differences in their resighting rates, we began by fitting the same model (ϕ ~ category + bSpline(Time), *p* ~ category + bSpline(Time)) to the mark‐recapture data from each year. Here bSpline(Time) is expected to deal with seasonal variation in weather conditions that may affect survival and resighting probability, but not age directly (since individuals were marked throughout the season).

Having achieved an overview, we then sought to fit tailor‐made models to each year's mark‐recapture data so that we could uncover consistent predictors. Given the large number of static and time‐varying predictors, we identified candidate models using automated stepwise model comparison procedures based on AIC. With forward stepwise selection we started with the intercept‐only model (*ϕ* ~ 1, *p* ~ 1) which estimated a single fixed resighting and survival parameter. We then identified the predictor which, if added to the formula for either *ϕ* or *p*, would reduce the AIC by the greatest amount. Once identified, we added this predictor to *ϕ* or *p* and then sought to identify which remaining predictor, if added to the existing model, would lower the AIC the most. The process was continued until no candidate predictor when added to the model reduced the AIC, at which point the simpler model was retained. Backward stepwise selection followed a similar approach but this time we started with the most complex model (*ϕ* ~ category + bSpline(Age) + bSpline(size) + bSpline(AvTemp) + bSpline(Rain) + bSpline(Sun); *p* ~ category + bSpline(Age) + bSpline(size) + bSpline(AvTemp) + bSpline(Rain) + bSpline(Sun)) and stripped off a single predictor from either *ϕ* or *p* that reduced the model's AIC by the greatest amount. The selection procedure was continued until no predictor when removed resulted in a model with lower AIC.

## 3 Results

**FIGURE 1:**
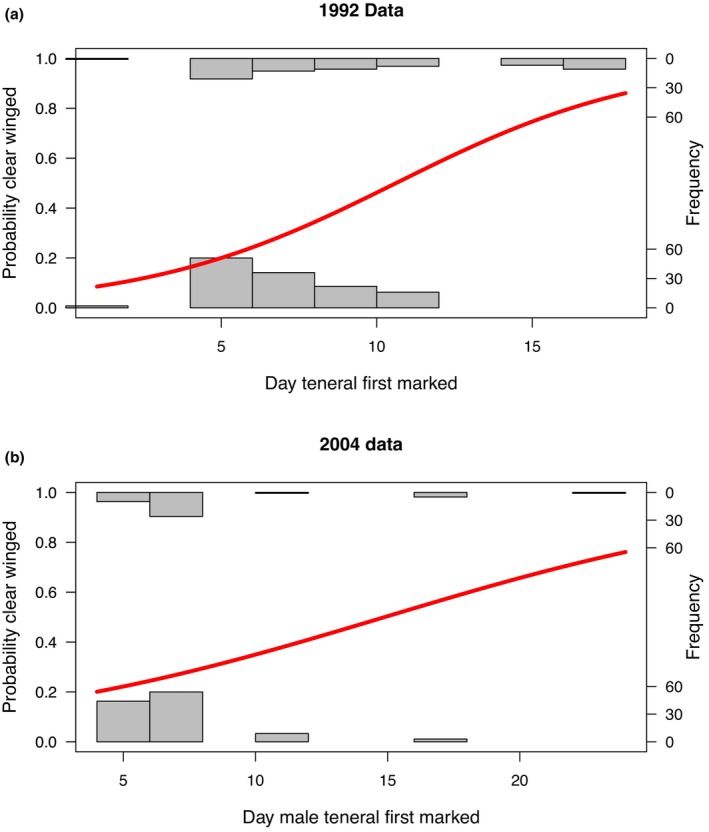
(a, b) Fit of a binary logistic model (red line) as to whether a pre‐reproductive individual marked on a given day was clear‐winged (1) or orange‐winged (0). Here, Day 0 represents the start of the experiment. In both (a) the 1992 MRR data set and (b) the 2004 MRR data set, the later a pre‐reproductive individual was caught and marked in the season the more likely it was to be clear‐winged.

We were first interested to know whether the emergence patterns of orange and clear‐winged morphs differed over the season. To do this we focused on the timing of first marking of pre‐reproductive males in 1992 and 2004 which had the highest sample size of both male morphs (> 40, Table 1). Binary logistic models (Figure 1a,b) indicated that newly marked pre‐reproductive males were significantly more likely to be clear‐winged as the season progressed (1992 logit slope = 0.247, *z* = 4.626, *p* < 0.001; 2004 logit slope = 0.127, *z* = 2.354, *p* = 0.019).

**FIGURE 2:**
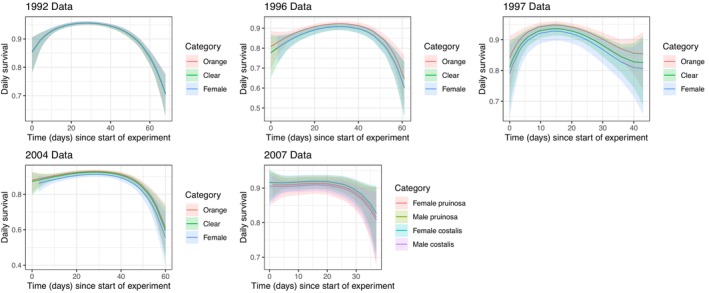
The estimated effect of category (orange‐winged male, clear‐winged male, female) and time in the season on the daily survival of marked adults (with 95% confidence intervals) following the fit of the model (ϕ ~ category + bSpline(Time), *p* ~ category + bSpline(Time)). Here, category was treated as a factor, while the changes in survival over the season were estimated using a polynomial spline. There was little evidence that daily survival varied with the category of damselfly. The survival curves for the categories overlap so closely in some years (e.g., 1992) that they appear as a single line.

Second, we wished to compare the survival of the male morphs while controlling for any differences in their resighting rates. Figure 2 shows the estimated survival (with 95% confidence limits) of the fit of the model (ϕ ~ category + bSpline(Time), *p* ~ category + bSpline(Time)) to each of the five data sets. Daily survival was typically highest in the middle of the season and tended to be lower at the start and end of the season. There was little evidence of a difference in daily survival between male morphs or sexes at a given time in the season once differences in their resighting rates were taken into account.

**FIGURE 3:**
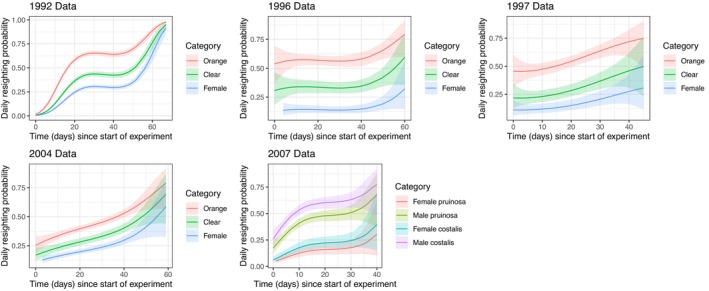
The estimated effect of category (orange‐winged male, clear‐winged male, female) and time in the season on the resighting probability of marked adult damselflies (with 95% confidence intervals) following the fit of the model (ϕ ~ category + bSpline(Time), *p* ~ category + bSpline(Time)). Here, category was treated as a factor, while the effect of time in the season on resighting probability was estimated using a polynomial spline. In contrast to survival, there was consistent and strong evidence that orange‐winged morphs were more detectable to researchers than clear‐winged morphs.

Figure 3 shows the jointly estimated resighting rates (with 95% confidence limits) from the fit of the above model. Resighting rates tended to increase as the season progressed, and there was a consistent difference in resighting between categories with orange‐winged males being more readily detected than clear‐winged males which were in turn more readily detected than females. To elucidate whether the low resighting rates at the start of the season were driven by a stronger tendency of individuals marked at the start of the season never to be seen again (“floaters,” Tsubaki et al. 1997) we fitted a series of logistic models. In 1992 and 2004, there was no significant relationship between the probability of an adult being seen again or not after first capture (coded as 1 or 0, respectively) and the time in the season it was first marked. However, in 1996, 1997, and 2007 individuals were more likely to be seen again the earlier in the season they were marked (1996 logit slope = −0.0121, *z* = −2.422, *p* = 0.015; 1997 logit slope = −0.0416, *z* = −4.319 *p* < 0.0001; 2007 logit slope = −0.0363, *z* = −3.249, *p* = 0.0012). Despite the overall increase in daily resighting rates, individuals marked late in the season will have had less opportunity to be resighted before the study was terminated. We consider how this truncation may affect the estimated survival rates based on minimum lifespan in our Supporting Information.

In contrast to its effect on survival, category was clearly an important predictor of resighting rates. To illustrate the importance of controlling for category‐dependent resighting rates when estimating survival, Figure S2 shows the fits of (ϕ ~ category + bSpline(Time), *p* ~ bSpline(Time)). After fitting this model, category had a more profound effect on daily survival rates, since with a single overall rate of detection of individuals assumed, highly detectable forms will be presumed to have higher survival compared to less detectable forms. Indeed, in every year except 1992 the model (ϕ ~ category + bSpline(Time), *p* ~ bSpline(Time)) had a far lower AIC than the model (ϕ ~ bSpline(Time), *p* ~ bSpline(Time)), with the apparent survival consistently being highest for orange‐winged males.

**FIGURE 4:**
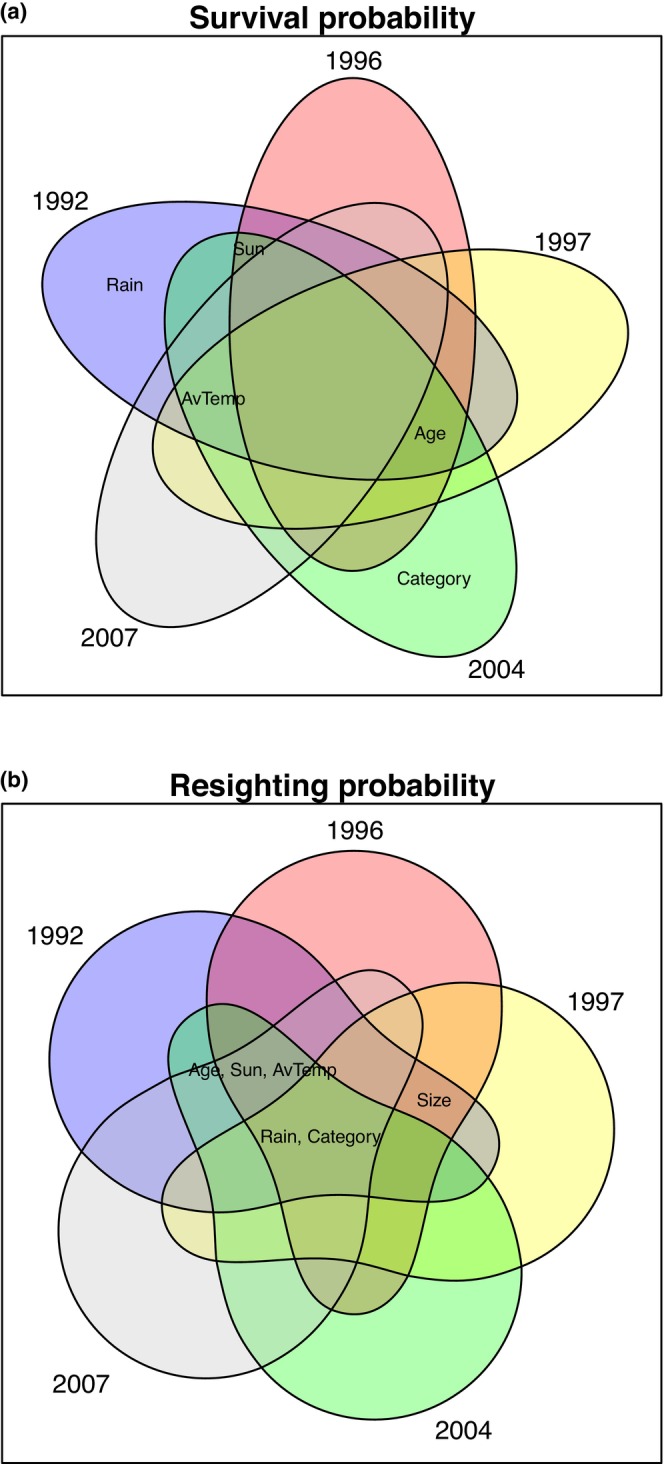
(a, b) Venn diagrams showing the predictors that were included in the most parsimonious model for (a) apparent survival and (b) resighting probability, based on forwards stepwise AIC over the five MRR studies (1992, 1996, 1997, 2004, 2007). Age and average temperature (both modeled as polynomial splines) were most consistently included in the most parsimonious model for daily survival, while category (orange‐winged, clear‐winged, female) and rainfall (modeled as a polynomial spline) were always included in the most parsimonious model for resighting probability.

Third, we wished to identify the models that best explained survival and resighting rates in each MRR data set, allowing different parsimonious models to be recognized for each field season. Table 2 shows the candidate model identified after fitting separate models to each year's data using forward automated stepwise model selection. The order of listing reflects the order in which they were added (see Tables S1–S5 for the orders in which the predictors were accumulated and lost in both directions). Different models were converged on through forwards and backwards iteration in our 1996 and 1997 data sets, but in both cases the model identified through forward iteration had the slightly lower AIC. Figure 4a,b shows the common predictors for survival and resighting in each of the five study years. The age of a damselfly (time from first capture) was a common predictor of its daily survival in all but one year (2007), while the average temperature was a common predictor of daily survival in all but one year (1996). Similarly, both the category of the damselfly and the amount of rainfall was a common predictor of daily resighting rates in all 5 years. Age was the first predictor to be added to explain variation in daily survival in three of the five field seasons. Category was the first predictor to be added to explain variation in resighting rates in four of the five field seasons. Hind wing size was never a significant predictor of survival, but it was included in the models for resighting in three of the five years.

**TABLE 2:** The lowest AIC models identified through automated forwards selection with six candidate predictors namely category (orange‐winged male, clear‐winged male, female), bSpline(Age), bSpline(size), bSpline(AvTemp), bSpline(Rain), and bSpline(Sun).

Year	Daily survival (*ϕ*)	Daily resighting rate (*p*)
1992	bSpline(Age) + bSpline(Sun) + bSpline(Rain) + bSpline(AvTemp)	bSpline(Rain) + category + bSpline(Age) + bSpline(Sun) + bSpline(AvTemp) + bSpline(size)
1996	bSpline(Sun) + bSpline(Age)	category + bSpline(AvTemp) + bSpline(size) + bSpline(Age) + bSpline(Sun) + bSpline(Rain)
1997	bSpline(Age) + bSpline(AvTemp)	category + bSpline(Rain) + bSpline(size)
2004	bSpline(Age) + bSpline(AvTemp) + bSpline(Sun) + category	category + bSpline(Sun) + bSpline(Rain) + bSpline(AvTemp) + bSpline(Age)
2007	bSpline(AvTemp)	category + bSpline(Sun) + bSpline(AvTemp) + bSpline(Rain) + bSpline(Age)

*Note:* The order in which the predictors are presented in each model is the order in which the predictors were accumulated in the model until no remaining predictor, when added, lowered the AIC. Forwards and backwards selection resulted in the same candidate model being identified in three of the five study years, and a similar but not identical model being identified in the remaining two study years (see Supporting Information for more details).

**FIGURE 5:**
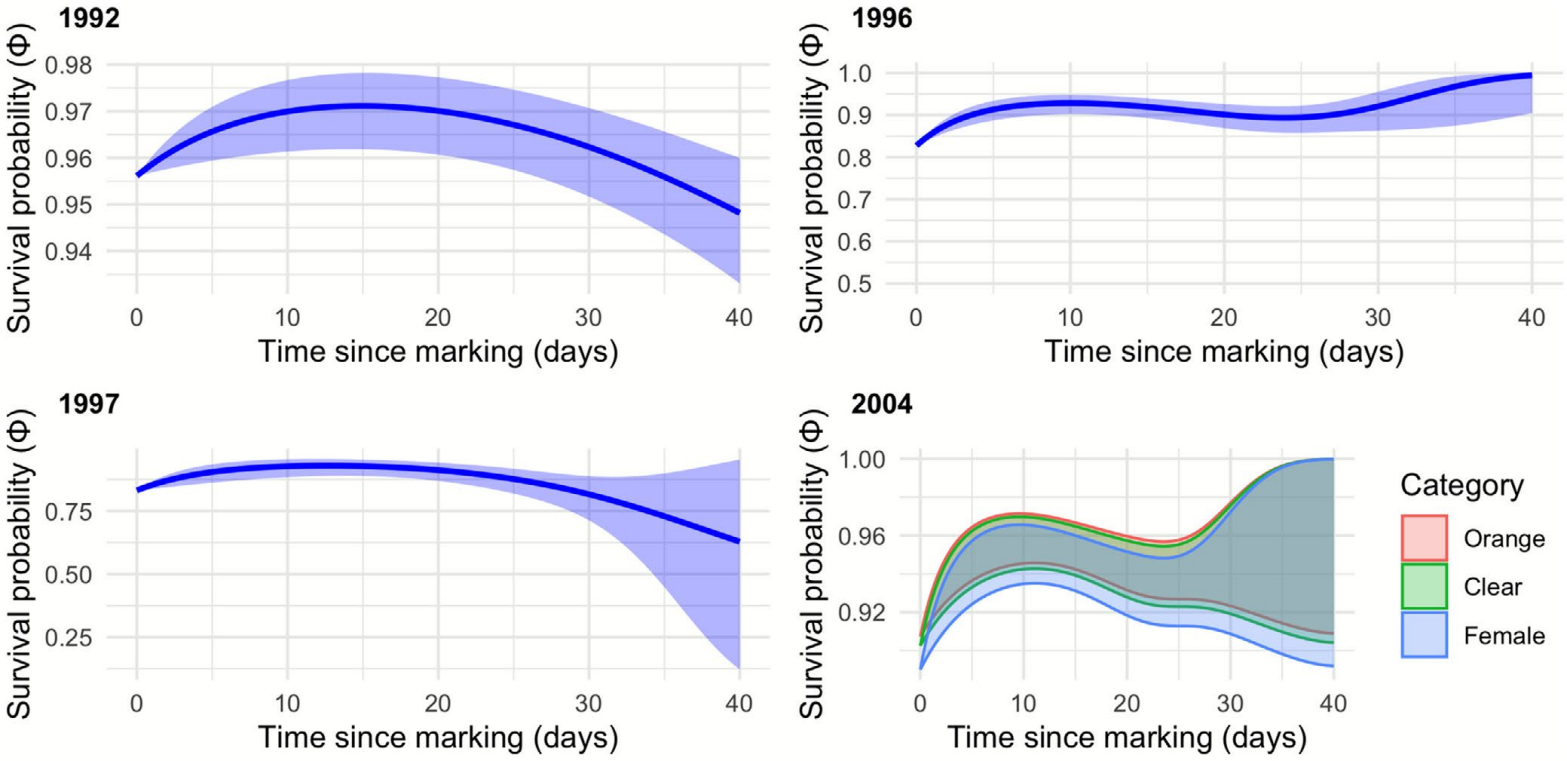
In four of the five study years, the time from which an individual was first marked (roughly speaking, age) was identified as a significant predictor of its daily survival (see Table 2). The plots here show the fit of the most parsimonious survival‐resighting model identified in each of these study years (with 95% confidence intervals), illustrating how daily survival changes with time since capture. For each year, all the remaining predictors in the most parsimonious model for that year were set at their mean values. In general, adult survival tends to rise for the first 10 days post capture and thereafter fall.

**FIGURE 6:**
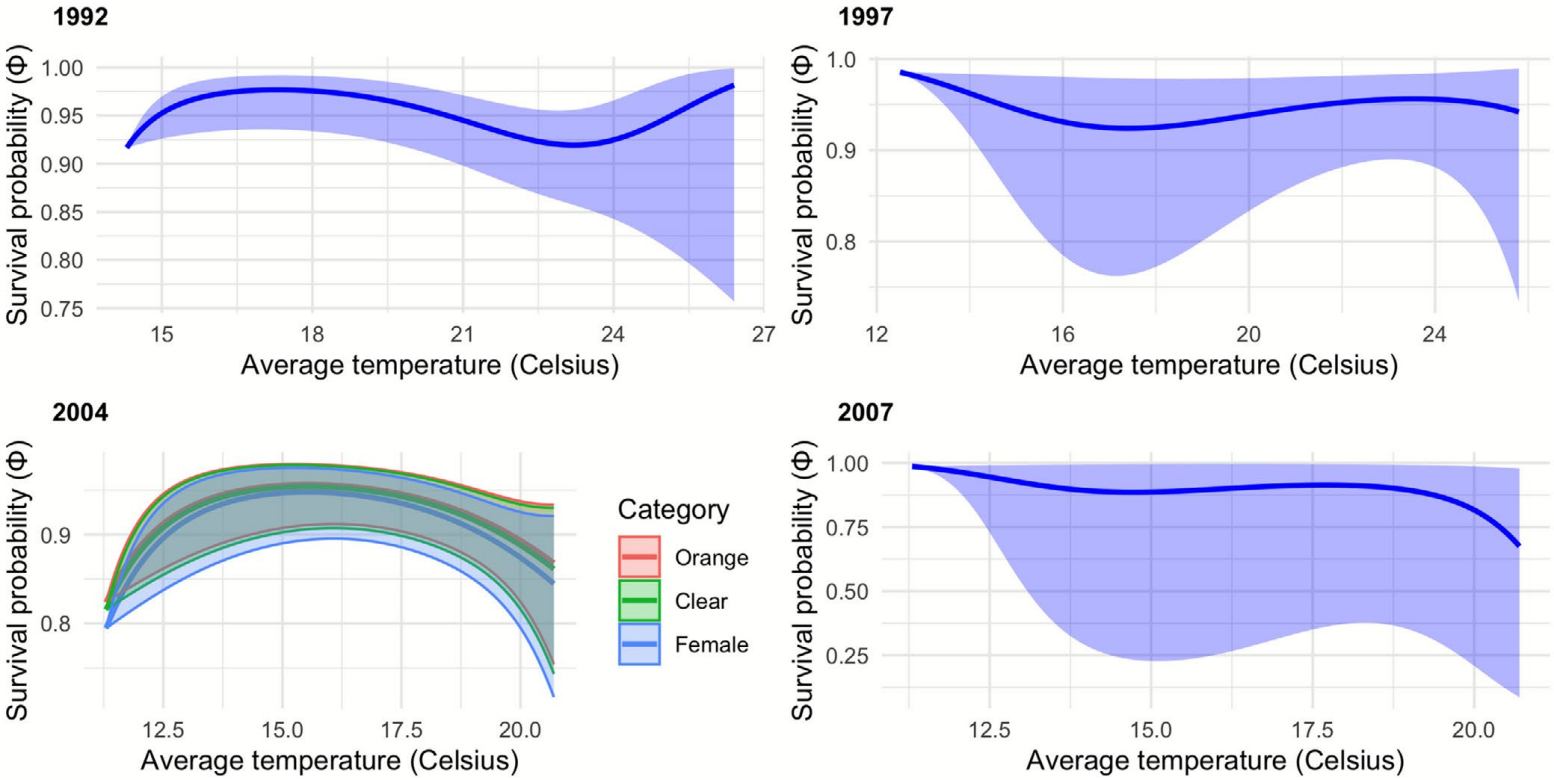
In four of the five study years, the average air temperature on an observation day was identified as a significant predictor of adult daily survival (see Table 2). The plots here show the fit of the most parsimonious model identified in each of these study years involving temperature (with 95% confidence intervals), illustrating how daily survival changes with average temperature. For each year, all the remaining predictors in the most parsimonious model for that year were set at their mean values. The fitted models differ between years but in general, adult survival tends to rise as the average temperature rises to approximately 17°C and thereafter falls.

Figure 5 shows the predicted changes in apparent daily survival with time since capture derived from the fits of the most parsimonious model for each of the four field seasons when time since capture (“Age”) was identified as a significant predictor (Table 2). In each case, the sizes of the other predictors remaining in the model were set to their mean (in 2004 category was identified as a predictor that lowered the AIC, so it is included as a predictor although there was high overlap in survival between categories). In three of the four years, the daily survival of adults following marking increased for about 10 days post capture and then fell off considerably. Figure 6 shows the predicted changes in daily survival with average temperature, with three of the four field seasons indicating a fall in survivorship as average air temperature increased beyond 17°C.

The relationship between daily resighting rates and rainfall was more complex (Figure S3), but this may be partly a consequence of model fitting. For example, in the 1992 data it only rained on 9 of the 43 days in which the site was visited, with no rain recorded between 7 and 20 mm, so the polynomial spline was free to interpolate in this range. Despite this, rain was still retained as a predictor for *p* when rain was coded as a binary predictor (0—no rain, 1—rain) following forwards and backwards stepwise model selection. Overall, the data sets roughly suggest resighting rates tend to increase up to around 15–20 mm of rain and then decline (note the low rainfall in 1996; the 2007 plot is not included because it only rained on 2 days creating high uncertainty).

Finally, we restricted model selection to just individuals marked when they were pre‐reproductive (so that their age at capture is indeed approximately 0) in the 1992 and 2004 data sets. In the 1992 data set, forwards and backwards stepwise regression resulted in the same model. This model was the same as that listed for all adults from 1992 in Table 2 but did not include temperature as a predictor of daily survival and did not include sunshine as a predictor of resighting rate. Category was the first variable to be dropped as a predictor of survival in backward model selection and the second to be included as a predictor of resighting in forwards model selection. In the 2004 pre‐reproductive data set, forwards and backwards model selection generated different models. In both cases, however, the most parsimonious model for survival involved both age and average temperature (implemented via a polynomial spline) but not category. The most parsimonious model for resighting involved category whether the model was identified via forwards or backwards selection. Category was the first to be dropped in backward model selection for survival and the first to be included as a predictor of resighting in forwards model selection.

## 4 Discussion

Alternate male reproductive tactics are widespread in the animal kingdom (Taborsky et al. 2008). Here, we have analyzed a series of mark‐recapture experiments on *Mnais* damselflies to assess the strength of evidence that orange‐winged (predominantly territorial) males survive at a lower rate than clear‐winged (predominantly sneaker) males. Our primary approach was to use CJS models to jointly estimate the survival and resighting rate of male morphs. We identified the primary drivers of survival and resighting by recognizing significant candidate predictors within a given study year, but also by highlighting those that were common across study years. After controlling for resighting rate, there was no clear evidence of a difference in survival between the two male morphs, or between males and females in any year. Not surprisingly, if we do not condition on resighting rates then forms with higher detection rates were judged to have significantly higher survival.

Many damselfly species, notably Coenagrionids, exhibit female‐limited polymorphism but there is no convincing evidence that the female morphs differ in survivorship, once differences in their resighting rates are accounted for (Sherratt et al. 2010; Sanmartín‐Villar and Cordero‐Rivera 2022). It is perhaps more surprising that there was no significant difference in the survival rate between the sexes of *Mnais costalis* given their very different behavior. Perhaps the extent of predation is not sufficiently different to render differences in survival between the sexes in *Mnais*. Predation by the Japanese wagtail ( *Motacilla grandis* ) has been observed, particularly after a heavy rain, but the wing remains left behind do not suggest high degrees of selectivity. Likewise, individuals have been seen to be attacked by the semi‐aquatic spider, *Dolomedes saganus* but these events appear opportunistic (Y.T., personal observations). Intriguingly, a lack of a sex difference in survivorship has also been reported in other well‐replicated studies on damselflies (Allen and Thompson 2014; Cordero‐Rivera et al. 2019). Moreover, a recent meta‐analysis of 39 mark‐recapture studies on odonates conducted by Sanmartín‐Villar and Cordero‐Rivera (2022) indicated there was no evidence of an effect of sex (whether part of an interaction or not) on survival.

The lack of a clear difference in survival between male morphs is also consistent with Nomakuchi et al. (1988) and with the analysis Tsubaki et al. (1997) when they assumed that *Mnais costalis* damselflies were of age 0 at the time of capture. In the Supporting Information, we provide proof that using the time between the last sighting and marking to generate survival curves provides a good approximation of true survival when the population under consideration is limited to those individuals that were resighted at least once (as Tsubaki et al. 1997 did). However, the approach can also underestimate true survival when resighted adults still alive at the end of the experiment are assessed as having shorter lives than they otherwise would. Classically, when fitting CJS models only intervals with data are used for estimating survival and detection probabilities, so these models do not make assumptions beyond the end of the study. Intriguingly, Sanmartín‐Villar and Cordero‐Rivera (2022) found that, over all species evaluated in their meta‐analysis, the estimated survival rate of both sexes was positively correlated with the duration of the study, ranging from a few days to over 100 days, but we cannot rule out the possibility that the length of the experiment was tailored to the longevity of the species being observed.

Tsubaki et al. (1997) did find evidence of a difference in the cumulative survival curves of the two different *Mnais* morphs when they assumed that all individuals emerged on 1st June (the final day a teneral was seen). The analysis we present here provides a possible alternative explanation for their result. Specifically, pre‐reproductive clear‐winged individuals tended to be first captured slightly later in the season than orange‐winged males (on average 2.61 days later from individuals caught as teneral in the 1992 data). So, choosing a fixed date of emergence would likely involve comparing the survival of reproductively younger clear‐winged males with that of older orange‐winged males. Declines in survival with age are now well established in damselflies (Sanmartín‐Villar and Cordero‐Rivera 2022; Youcefi et al. 2024) including *Mnais* (see below), so the net effect of comparing the survival of individuals as if they all emerged on the same day may give the impression that the later maturing clear‐winged males subsequently live longer than orange‐winged males.

Rearing experiments on *Mnais* have led to similar mixed results that could also, on reflection, be understood at least in part as a consequence of the different phenologies of the two morphs. In particular after randomly catching mature males of each morph and maintaining them in the lab, there were significant differences in the survival curves of the morphs when they were caught late in the season (i.e., mid‐June onwards) (Tsubaki et al. 1997; Tsubaki and Hooper 2004), but not when caught early in the season (May and early June) (Tsubaki and Hooper 2004). Condition dependence in which field‐caught orange males were more exhausted than clear males when captured in late season may have played a role here (Tsubaki and Hooper 2004). However, given that randomly caught males are more likely to be similar in age when captured at the start of the season compared to later, there is an obvious need to conduct analogous experiments using tenerals of known age.

One might wonder why the male morphs would show differences in their phenology. In the 1992 data for example, approximately twice as many orange teneral males than clear‐winged teneral males were marked in mid‐May, but a smaller and similar number of both forms were marked in late May (Tsubaki et al. 1997). If detectability affected the perceived emergence times of the two male morphs, then one might expect clear‐winged males to appear as though they emerge late and cease to emerge earlier, which has not been reported. In a 10‐year study investigating the possibility of protandry in *Mnais*, Watanabe and Taguchi (2000) noted that while there was some overlap, the average date at which no new emergences were observed was early June for orange‐winged males; yet it was late June for clear‐winged males and females, indicating that orange‐winged males have a shorter emergence period. Moreover, mature orange‐winged males were seen at the stream sooner than those of the clear‐winged males (and females) in every year of the decade they were observed. As Watanabe and Taguchi (2000) argue, the tendency of orange‐winged males to arrive sooner at streams makes biological sense: orange‐winged males may face selection to secure good territories before others get to them, while clear‐winged males may face selection to arrive later since they tend to gain by exploiting the territorial behavior of established orange‐winged males.

Studies comparing the lifespan of male insects with territorial and sneaker tactics are relatively rare. Kotiaho and Simmons (2003) found no difference in lifespan between conditional alternative mating tactics in the beetle *Onthophagus binodis* . Although Sato et al. (2016) found that young sneaker males of the two‐spotted spider mite (*Tetranychus urticae*) survived longer than young fighter males under mild male competition, this difference was not evident under strong male competition. The “reproductive lifespan” (time of last appearance—time of marking) of male *Calopteryx maculata* that were observed sneaking copulations at some time in their lives was longer than that of territorial males who were not observed sneaking (Forsyth and Montgomerie 1987). However, the sneaker strategy is flexible in this species, with previously territorial males making “the best of bad situation” by becoming sneakers late in life (Forsyth and Montgomerie 1987; Tsubaki and Ono 1987; Waltz and Wolf 2015).

While our focus was on comparing the survival rates of the two morphs, we also wished to elucidate the predictors that consistently best explained survival and resighting. The age (or, more accurately, its time since first capture) of a damselfly was a predictor of its daily survival in all but 1 year, including individuals marked when pre‐reproductive. The survival rate of individuals initially following capture appears low, possibly because of unintentional damage inflicted on individuals when netted, handled and marked but more likely because immature damselflies tend to have lower survival than mature damselflies (Anholt 1991; Cordero 1994; Allen and Thompson 2014). Not surprisingly, the survival of marked adults tended to be lower in individuals of advanced age, likely as a result of accumulated damage (males at the end of the season regularly appear with tattered wings) and physiological decline (Sherratt et al. 2010; Allen and Thompson 2014; Hassall et al. 2015; La Porta and Goretti 2019; Sanmartín‐Villar and Cordero‐Rivera 2022; Youcefi et al. 2024). Average air temperature was also a common predictor of survival, having a negative effect on survival beyond approximately 17°C. In support, Tsubaki and Samejima (2016) found that the minimum reproductive lifespan (i.e., the time difference between last and first sighting) of territorial *M. costalis* males declined significantly with the openness of the canopy in their territories. High temperatures promote increased activity (Tsubaki et al. 2010) which may result in the rapid depletion of fat reserves leading to exhaustion (Plaistow and Tsubaki 2000). In contrast to air temperature, the amount of rainfall per day was not a strong predictor of the daily survival of *Mnais* damselflies, most likely because individuals shelter when wet by perching within tree canopies. As sheltering individuals do not fly around or interact with others, they most likely conserve their energy for the next day.

Throughout all our analyses, the orange‐winged predominantly territorial males were resighted more readily than the clear‐winged males, which were in turn resighted more frequently than females, consistent with the findings of MunguÍa‐Steyer et al. (2010) in which the territorial male morph was more detectable. This makes intuitive sense based on both the morphology and behavior of the morphs, with conspicuous orange‐winged males actively defending sunny oviposition sites and females only turning up at these territories when it is time to mate. In all years, the daily resighting rates of females and both male morphs tended to increase, sometimes dramatically, as the season progressed. Pre‐reproductive individuals typically spend much of their time away from the stream to feed and develop. As they mature, they will spend less time feeding and more time at the stream, eventually dedicating much of their final time to reproduction. Tsubaki et al. (1997) reported that the proportion of clear males in the population increased as the season progressed. Although this observation is consistent with the possibility that clear‐winged males live longer, we must now also consider the fact that clear‐winged males tend to arrive at streams slightly later in the season and the fact that both morph types are more frequently resighted at the stream as the season progresses.

In the 2007 data, orange‐winged male *M. costalis* were more readily detected than clear‐winged *M. pruinosa* but both species of male had higher resighting probabilities than females of their own species, reinforcing the fact that sex is even more important than morph in shaping resighting rates. One might expect that the resighting rates of *M. costalis* females would be higher when they are sympatric with *M. pruinosa* than when the species is found alone, because in the former case there are no conspecific sneakers to harass them. There is little evidence of a difference from our analysis, although it is challenging to compare resighting rates between years when other factors such as weather, canopy openness and the number of observers differ between field seasons.

Damselflies are readily caught and marked and do not tend to disperse far from where they emerge (Conrad et al. 1999) which makes them highly suitable for classical mark‐release‐recapture (MRR) analyses (Anholt et al. 2001; Cordero‐Rivera and Stoks 2008; Sherratt et al. 2010; Sanmartín‐Villar and Cordero‐Rivera 2022; Youcefi et al. 2024). Reassuringly the rate of movement of *Mnais* damselflies appears relatively low. For example, Suzuki and Tamaishi (1981) conducted an intensive mark and recapture study of *M. costalis* adults along a stream and found that the average movement distance of resighted *M. costalis* per day was 15.0 m (male: 16.4 m, female: 10.0 m). In our Figure S4, we show the distribution of the average displacement rates per day of resighted males and females in our 1992 data. The displacement rates per day are similar to the distances reported in this earlier study for males (mean displacement per day orange‐winged males 14.0 m, clear‐winged males 22.5 m), although we found that females at our study site dispersed more widely (33.1 m per day for females). One might expect that orange‐winged males would remain at a given territory for as long as they can maintain it, while clear‐winged males are more opportunistic and move further afield in search of females, which is precisely what we see here.

If movement takes individuals temporarily away from the search area, then so long as all individuals of a given type have the same probability of being absent on a given day when the search takes place then the estimates of their survival will be unbiased (Schaub et al. 2004). If however the probability of an individual being temporarily away from the search area affects their probability of being absent on the next search (the probabilities are “non‐Markov”) then it can lead to biased survival estimates (Kendall et al. 1997). Most concerning, if some of this movement takes individuals permanently away from the search area during the study then it can have an important effect on the estimates of survival. The reason for this is simple: most standard MRR analyses quantify “apparent survival,” which is the probability that an individual survives between search occasions and returns to the sampling area (Lebreton et al. 1992; Sandercock 2020). If movement results in a higher tendency of one of the morphs (or sexes) to permanently leave the study area, then it could lead to a bias when comparing their survivorship. Spatially explicit capture–recapture (SECR) models (Baird et al. 2012; Borchers 2012; Ergon and Gardner 2014; Schaub and Royle 2014; Efford and Schofield 2022; Badia‐Boher et al. 2023; Birch et al. 2025) offer the possibility of jointly modeling movement (including permanent emigration) via a diffusion process, resighting and survival, and could provide the basis of an even more sophisticated analysis in the future.

Unlike many other calopterygid damselflies, the male wing color of *Mnais* is under genetic control (Tsubaki 2003). Genetically fixed ARTs are more likely to evolve when the mating skew is high, so that very high degrees of specialism are needed to ensure paternity rather than a more flexible, jack‐of‐all‐trades strategy (Plaistow et al. 2004). It should be stressed that survival is not always the key determinant of lifetime reproductive success in these systems (Banks and Thompson 1987). Indeed, the detailed field observations of Tsubaki et al. (1997) on *Mnais costalis* suggested that the mean number of days males were reproductively active was a fraction of the estimated lifespans of these morphs, with orange‐winged males holding territories for a limited time compared to the sneaking activities of clear‐winged. So, it appears that what a morph does with its time is more important than how long it lives.

## Author Contributions

**Yoshitaka Tsubaki:** conceptualization (lead), data curation (lead), funding acquisition (lead), investigation (lead), methodology (lead), project administration (lead), supervision (lead), writing – review and editing (equal). **Rowan E. Hooper:** conceptualization (lead), investigation (lead), methodology (lead), writing – review and editing (equal). **Stewart Plaistow:** conceptualization (lead), investigation (lead), methodology (lead), writing – review and editing (equal). **Yuka Samejima:** conceptualization (lead), investigation (lead), methodology (lead), writing – review and editing (equal). **Thomas N. Sherratt:** data curation (equal), formal analysis (lead), funding acquisition (equal), visualization (lead), writing – original draft (lead), writing – review and editing (equal).

## Funding

This work was supported by the Natural Sciences and Engineering Research Council of Canada.

## Disclosure

Diversity Statement: Our study brings together male and female authors from three different countries with complementary expertise in odonate biology and modeling, including scientists based in Japan where the study was carried out. Whenever relevant, literature published by scientists from Japan is cited, including papers published in Japanese.

## Conflicts of Interest

The authors declare no conflicts of interest.

## Supporting information


**Appendix S1:** ece372623‐sup‐0001‐AppendixS1.docx.

**Data S1:** ece372623‐sup‐0002‐DataS1.zip.

## Data Availability

Our mark‐recapture data, meterological data and R code for stepwise regression modelling are available as Supporting Information.
