# Supplementary material for: Similar Survival Rates of Territorial and Sneaker Males in a Polymorphic Damselfly: A Multi‐Year Study
Source: Ecol Evol. 2025 Dec 10;15(12):e72623. doi: 10.1002/ece3.72623 (PMC12690216; doi:10.1002/ece3.72623)
Supplement: Supplementary file 1 — Appendix S1: ece372623‐sup‐0001‐AppendixS1.docx. [file ECE3-15-e72623-s002.docx]

**Supporting Information**

Similar survival rates of territorial and sneaker males in a polymorphic damselfly: a multi-year study

**Using the time between last sighting and first marking to derive survival curves**

We begin by deriving the distribution of the time in days between last resighting and first capture of a large population of marked damselflies. We assume for computational convenience that all the damselflies are marked on day 0 and that the experiment runs for *T* days, after which time marked damselflies are not searched for. We assume that the damselflies have an overnight survival probability of *s* and that the probability of a marked individual being seen by a researcher on any given day if they are alive is *r*. Dead individuals are never seen.

We seek the probability that the last resighting of a marked individual occurs on day *d*, where *d* = 0,1…*T.* Formally, let *L* be the last day an individual marked damselfly is seen (i.e. its last resighting) with Pr(*L* = *d*) reflecting the probability that the last sighting of a marked damselfly was on day *d*. For an individual to be last seen on day *d* then it must be alive and seen on day *d* but it also must not be seen on the following days *d*+1, *d*+2...*T*. Individuals will not be seen on a given day after day *d* either because they are dead or because they are alive and not resighted.

The probability of a marked damselfly being alive and seen on day *d* is simply ${r s}^{d}$. The probability that this resighted individual is never seen again after day *d* is harder to calculate but can be derived through recursion. Thus, let us define *A_j_* as the probability that an individual alive on day *j* is never again seen after that day (i.e. any day *j*+1 up to *T*)*.* Whether seen or unseen on day *j*, the damselfly could die overnight between day *j* and day *j* + 1 with probability (1-*s*) or survive overnight and not be seen the next day (day *j*+1) with probability *s* (1-*r*). If the damselfly survives overnight but is not seen the next day, the calculation repeats for this next day. For day *j* $\leq$ *T* we know that:

$$A_{j}=\left( 1-s \right)+s\left( 1-r \right) A_{j+1}$$

where $A_{j+1}$is the probability that, given the individual’s survival to day *j*+1, it is never seen again. A boundary condition $A_{T}=1, \mathrm{applies}$since marked damselflies are certain not to be seen after the experiment has ended (even if some are still alive). The above equation relating $A_{j}$ to $A_{j+1}$is a first order recursion equation that has an explicit solution namely:

$$A_{j}=(1-s)\frac{1- {(s\left( 1-r \right))}^{T-j}}{1-s(1-r)}+{(s\left( 1-r \right))}^{T-j}$$

Putting these components together, the probability that the last sighting is on day *d* is the probability of being alive on day *d* times the probability of being resighted on day *d* if alive, times the probability of never being seen again after day *d*, namely:

$$\Pr\left( L=d \right)=r s^{d}A_{d}$$

The probability that the last sighting is on day *d* is therefore:

$$\Pr\left( L=d \right)=rs^{d}((1-s)\frac{1- {(s\left( 1-r \right))}^{T-d}}{1-s(1-r)}+{(s\left( 1-r \right))}^{T-d})$$

We now seek to calculate the cumulative survival function based on the population of marked individuals that were seen at least once (i.e. the resighted population). The proportion of all marked individuals that are seen one or more times during the course of the experiment is:

$$\sum_{x=1}^{T} \Pr\left( L=x \right)=\sum_{x=1}^{T} (rs^{x}((1-s)\frac{1- {(s\left( 1-r \right))}^{T-x}}{1-s(1-r)}+{(s\left( 1-r \right))}^{T-x}))$$

To generate the cumulative survival, we simply need to calculate the proportion of the resighted population that survived for 1 day or more, 2 days or more etc. This is given by:

$$S\left( d \right)=\frac{\sum_{x=d}^{T} \Pr\left( L=x \right)}{\sum_{x=1}^{T} \Pr\left( L=x \right)}$$

so that *S*(1) = 1 but declines as *d* increases. This ratio simplifies to:

$$S\left( d \right)=\frac{s^{d}(\frac{1}{s}+(r-1){(s-rs)}^{T-d})}{1-{(s-rs)}^{T}}$$

Note that when *r* = 1 or *T* is large relative to the expected longevity (which is lower the lower *s*) then ${S\left( d \right)=s}^{d-1}$, which is the true cumulative survival function. The reason why the effect of *r* is small when *T* is large is that while the total number of individuals seen again is lower the lower *r*, it does not affect the shape of the distribution of the cumulative survival function based on observed longevities. However, when the experiment is of short duration i.e. *T* is low (or individuals continue to be caught and marked close to the end of the season) then there is a bias that is introduced (see **Figure S1**) which is more extreme if the marked individuals have low resighting rates. This bias arises because hard to detect individuals are more likely to be excluded when the experiment ends as a consequence of them not being seen, rather than through death.

**Figure S1.** Analytically derived survival curves for a cryptic and a conspicuous morph when we use the cumulative proportion of the resighted population that were seen on day 1 or later, day 2 or later etc. Model parameters, *s* = 0.95, *T* = 30. The use of time between last resighting and marking provides an excellent approximation of true survival (assuming survival and resighting rates are constant) so long as the experiment is terminated after all marked individuals are likely to have died. If the experiment is terminated early (or individuals are marked right up to the end of the experiment) then biases are introduced which lower the apparent survival in a manner that is more extreme when individuals are hard to detect.

**Figure S2**

**Figure S2.** The estimated effect of category (orange-winged male, clear-winged male, female) and time in the season on the daily survival probability of marked adult damselflies (with 95% confidence intervals) following the fit of the model (ϕ~ category + bSpline(Time), p~ bSpline(Time)) so that resighting rates in this model are no longer allowed to vary with category. In contrast to **Figure 2** in the main text where category is retained as a predictor of resighting rates, not controlling for the resighting rates of the different categories of damselfly can give the impression that more detectable forms have higher survival rates than less detectable forms. The survival curves for the categories overlap so closely in some years (notably 1992) that they appear as a single line.

**Figure S3**

**Figure S3.** In all the study years, the category (orange-winged male, clear-winged male, female) of adult and amount of rainfall were identified as significant predictors of the daily resighting rate of marked individuals (see Table 2). The plots show the fit of the most parsimonious survival-resighting model identified in each of these years (with 95% confidence intervals), illustrating how daily resighting rates vary in a manner that is dependent on category and rainfall. In each case, all the remaining predictors in any given model were set at their mean values. The 2007 data are not plotted because there was so little rainfall. Resighting rates were consistently highest for orange-winged territorial males and lowest for females. The probability of detecting adults of any form declined after 20mm of rainfall.

**Figure S4**

**Figure S4.** The estimated velocities (distances moved upstream or downstream per day) of marked damselflies observed in the 1992 field data. These calculations were based on individuals first caught on the stream (some were originally caught in bushes away from the water with no location) and never found elsewhere, so that displacements per unit time could be readily calculated. When several days passed between consecutive resighting, we simply calculated the displacement per unit day ($\Delta x$ / $\Delta t$). Orange-winged individuals tend to be territorial and were not seen to move very far from their territories once established, with a clear peak displacement per day of 0m. Clear winged males actively seek to exploit the territorial behaviour of orange-winged males and disperse a little more widely. Females were even more dispersive.

| FORWARDS ITERATION | | | | |
| --- | --- | --- | --- | --- |
| Iteration | *ϕ* | | *p* | AIC |
| 0 | 1 | | 1 | 15173.18 |
| 1 | 1 | | bSpline(Rain) | 14790.24 |
| 2 | 1 | | bSpline(Rain) + category | 14410.70 |
| 3 | 1 | | bSpline(Rain) + category + bSpline(Age) | 14292.51 |
| 4 | bSpline(Age) | | bSpline(Rain) + category + bSpline(Age) | 14186.16 |
| 5 | bSpline(Age) | | bSpline(Rain) + category + bSpline(Age) + bSpline(Sun) | 14097.54 |
| 6 | bSpline(Age) + bSpline(Sun) | | bSpline(Rain) + category + bSpline(Age) + bSpline(Sun) | 14063.52 |
| 7 | bSpline(Age) + bSpline(Sun) + bSpline(Rain) | | bSpline(Rain) + category + bSpline(Age) + bSpline(Sun) | 14012.74 |
| 8 | bSpline(Age) + bSpline(Sun) + bSpline(Rain) | | bSpline(Rain) + category + bSpline(Age) + bSpline(Sun) + bSpline(AvTemp) | 13978.22 |
| 9 | bSpline(Age) + bSpline(Sun) + bSpline(Rain) | | bSpline(Rain) + category + bSpline(Age) + bSpline(Sun) + bSpline(AvTemp) + bSpline(size) | 13957.59 |
| 10 | bSpline(Age) + bSpline(Sun) + bSpline(Rain) + bSpline(AvTemp) | | bSpline(Rain) + category + bSpline(Age) + bSpline(Sun) + bSpline(AvTemp) + bSpline(size) | 13929.29 |
|  | | | | |
| BACKWARDS ITERATION | | | | |
| Iteration | | *ϕ* | *p* | AIC |
| 0 | | category + bSpline(Age) + bSpline(size) + bSpline(AvTemp) + bSpline(Rain) + bSpline(Sun) | category + bSpline(Age) + bSpline(size) + bSpline(AvTemp) + bSpline(Rain) + bSpline(Sun) | 14562.70 |
| 1 | | bSpline(Age) + bSpline(size) + bSpline(AvTemp) + bSpline(Rain) + bSpline(Sun) | category + bSpline(Age) + bSpline(size) + bSpline(AvTemp) + bSpline(Rain) + bSpline(Sun) | 13931.99 |
| 2 | | bSpline(Age) + bSpline(AvTemp) + bSpline(Rain) + bSpline(Sun) | category + bSpline(Age) + bSpline(size) + bSpline(AvTemp) + bSpline(Rain) + bSpline(Sun) | 13929.29 |

**Table S1.** The most parsimonious models identified through automated forwards and backwards stepwise selection based on AIC when repeatedly fitted to the 1992 field data. There were six candidate predictors, as shown in the maximal backwards model at iteration 0. Forwards and backwards selection resulted in the same candidate model being identified.

| FORWARDS ITERATION | | | |
| --- | --- | --- | --- |
| Iteration | ϕ | p | AIC |
| 0 | 1 | 1 | 4985.04 |
| 1 | 1 | category | 4699.28 |
| 2 | bSpline(Sun) | category | 4667.59 |
| 3 | bSpline(Sun) | category + bSpline(AvTemp) | 4645.70 |
| 4 | bSpline(Sun) | category + bSpline(AvTemp) + bSpline(size) | 4628.46 |
| 5 | bSpline(Sun) + bSpline(Age) | category + bSpline(AvTemp) + bSpline(size) | 4614.46 |
| 6 | bSpline(Sun) + bSpline(Age) | category + bSpline(AvTemp) + bSpline(size) + bSpline(Age) | 4596.09 |
| 7 | bSpline(Sun) + bSpline(Age) | category + bSpline(AvTemp) + bSpline(size) + bSpline(Age) + bSpline(Sun) | 4588.88 |
| 8 | bSpline(Sun) + bSpline(Age) | category + bSpline(AvTemp) + bSpline(size) + bSpline(Age) + bSpline(Sun) + bSpline(Rain) | 4580.11 |
| BACKWARDS ITERATION | | | |
| Iteration | ϕ | p | AIC |
| 0 | category + bSpline(Age) + bSpline(size) + bSpline(AvTemp) + bSpline(Rain) + bSpline(Sun) | category + bSpline(Age) + bSpline(size) + bSpline(AvTemp) + bSpline(Rain) + bSpline(Sun) | 4587.82 |
| 1 | bSpline(Age) + bSpline(size) + bSpline(AvTemp) + bSpline(Rain) + bSpline(Sun) | category + bSpline(Age) + bSpline(size) + bSpline(AvTemp) + bSpline(Rain) + bSpline(Sun) | 4586.23 |
| 2 | bSpline(Age) + bSpline(size) + bSpline(AvTemp) + bSpline(Rain) | category + bSpline(Age) + bSpline(size) + bSpline(AvTemp) + bSpline(Rain) + bSpline(Sun) | 4585.37 |
| 3 | bSpline(Age) + bSpline(AvTemp) + bSpline(Rain) | category + bSpline(Age) + bSpline(size) + bSpline(AvTemp) + bSpline(Rain) + bSpline(Sun) | 4580.87 |

**Table S2.** The most parsimonious models identified through automated forwards and backwards stepwise selection based on AIC when repeatedly fitted to the 1996 field data. There were six candidate predictors. Forwards and backwards selection resulted in a different model being identified for survival but the same candidate model being identified for resighting.

| FORWARDS ITERATION | | | |
| --- | --- | --- | --- |
| IteratIon | ϕ | p | AIC |
| 0 | 1 | 1 | 2918.17 |
| 1 | 1 | category | 2762.53 |
| 2 | bSpline(Age) | category | 2747.19 |
| 3 | bSpline(Age) | category + bSpline(Rain) | 2735.21 |
| 4 | bSpline(Age) + bSpline(AvTemp) | category + bSpline(Rain) | 2726.93 |
| 5 | bSpline(Age) + bSpline(AvTemp) | category + bSpline(Rain) + bSpline(size) | 2720.86 |
|  | | | |
| BACKWARDS ITERATION | | | |
| Iteration | ϕ | p | AIC |
| 0 | category + bSpline(Age) + bSpline(size) + bSpline(AvTemp) + bSpline(Rain) + bSpline(Sun) | category + bSpline(Age) + bSpline(size) + bSpline(AvTemp) + bSpline(Rain) + bSpline(Sun) | 2742.96 |
| 1 | category + bSpline(Age) + bSpline(size) + bSpline(AvTemp) + bSpline(Rain) + bSpline(Sun) | category + bSpline(Age) + bSpline(size) + bSpline(AvTemp) + bSpline(Sun) | 2732.75 |
| 2 | category + bSpline(Age) + bSpline(AvTemp) + bSpline(Rain) + bSpline(Sun) | category + bSpline(Age) + bSpline(size) + bSpline(AvTemp) + bSpline(Sun) | 2728.70 |
| 3 | bSpline(Age) + bSpline(AvTemp) + bSpline(Rain) + bSpline(Sun) | category + bSpline(Age) + bSpline(size) + bSpline(AvTemp) + bSpline(Sun) | 2721.09 |

**Table S3** The most parsimonious models identified through automated forwards and backwards stepwise selection based on AIC when repeatedly fitted to the 1996 field data. There were six candidate predictors. Forwards and backwards selection resulted in different models being identified for survival and resighting but with similar AIC.

| FORWARD ITERATION | | | |
| --- | --- | --- | --- |
| Iteration | ϕ | p | AIC |
| 0 | 1 | 1 | 9259.06 |
| 1 | 1 | category | 9087.64 |
| 2 | bSpline(Age) | category | 9045.21 |
| 3 | bSpline(Age) | category + bSpline(Sun) | 9022.94 |
| 4 | bSpline(Age) | category + bSpline(Sun) + bSpline(Rain) | 8986.23 |
| 5 | bSpline(Age) | category + bSpline(Sun) + bSpline(Rain) + bSpline(AvTemp) | 8969.98 |
| 6 | bSpline(Age) + bSpline(AvTemp) | category + bSpline(Sun) + bSpline(Rain) + bSpline(AvTemp) | 8965.52 |
| 7 | bSpline(Age) + bSpline(AvTemp) + bSpline(Sun) | category + bSpline(Sun) + bSpline(Rain) + bSpline(AvTemp) | 8957.80 |
| 8 | bSpline(Age) + bSpline(AvTemp) + bSpline(Sun) | category + bSpline(Sun) + bSpline(Rain) + bSpline(AvTemp) + bSpline(Age) | 8955.62 |
| 9 | bSpline(Age) + bSpline(AvTemp) + bSpline(Sun) + category | category + bSpline(Sun) + bSpline(Rain) + bSpline(AvTemp) + bSpline(Age) | 8955.09 |
|  | | | |
| BACKWARDS ITERATION | | | |
| Iteration | ϕ | p | AIC |
| 0 | category + bSpline(Age) + bSpline(size) + bSpline(AvTemp) + bSpline(Rain) + bSpline(Sun) | category + bSpline(Age) + bSpline(size) + bSpline(AvTemp) + bSpline(Rain) + bSpline(Sun) | 8962.13 |
| 1 | category + bSpline(Age) + bSpline(AvTemp) + bSpline(Rain) + bSpline(Sun) | category + bSpline(Age) + bSpline(size) + bSpline(AvTemp) + bSpline(Rain) + bSpline(Sun) | 8958.29 |
| 2 | category + bSpline(Age) + bSpline(AvTemp) + bSpline(Rain) + bSpline(Sun) | category + bSpline(Age) + bSpline(AvTemp) + bSpline(Rain) + bSpline(Sun) | 8957.57 |
| 3 | category + bSpline(Age) + bSpline(AvTemp) + bSpline(Sun) | category + bSpline(Age) + bSpline(AvTemp) + bSpline(Rain) + bSpline(Sun) | 8955.09 |

**Table S4** The most parsimonious models identified through automated forwards and backwards stepwise selection based on AIC when repeatedly fitted to the 2004 field data. There were six candidate predictors. Forwards and backwards selection resulted in the same candidate joint model being identified for survival and resighting.

| FORWARDS ITERATION | | | |
| --- | --- | --- | --- |
| Iteration | ϕ | p | AIC |
| 0 | 1 | 1 | 5006.63 |
| 1 | 1 | category | 4833.64 |
| 2 | 1 | category + bSpline(Sun) | 4699.38 |
| 3 | 1 | category + bSpline(Sun) + bSpline(AvTemp) | 4613.54 |
| 4 | 1 | category + bSpline(Sun) + bSpline(AvTemp) + bSpline(Rain) | 4553.76 |
| 5 | 1 | category + bSpline(Sun) + bSpline(AvTemp) + bSpline(Rain) + bSpline(Age) | 4526.15 |
| 6 | bSpline(AvTemp) | category + bSpline(Sun) + bSpline(AvTemp) + bSpline(Rain) + bSpline(Age) | 4524.55 |
| BACKWARDS ITERATION | | |  |
| Iteration | ϕ | p | AIC |
| 0 | category + bSpline(Age) + bSpline(AvTemp) + bSpline(Rain) + bSpline(Sun) | category + bSpline(Age) + bSpline(AvTemp) + bSpline(Rain) + bSpline(Sun) | 4540.72 |
| 1 | category + bSpline(Age) + bSpline(AvTemp) + bSpline(Sun) | category + bSpline(Age) + bSpline(AvTemp) + bSpline(Rain) + bSpline(Sun) | 4534.23 |
| 2 | bSpline(Age) + bSpline(AvTemp) + bSpline(Sun) | category + bSpline(Age) + bSpline(AvTemp) + bSpline(Rain) + bSpline(Sun) | 4530.40 |
| 3 | bSpline(AvTemp) + bSpline(Sun) | category + bSpline(Age) + bSpline(AvTemp) + bSpline(Rain) + bSpline(Sun) | 4527.05 |
| 4 | bSpline(AvTemp) | category + bSpline(Age) + bSpline(AvTemp) + bSpline(Rain) + bSpline(Sun) | 4524.55 |

**Table S5**. The most parsimonious models identified through automated forwards and backwards stepwise selection based on AIC when repeatedly fitted to the 2007 field data. There were six candidate predictors, including category with 4 levels (males and females of both species). Forwards and backwards selection resulted in the same candidate joint model being identified for survival and resighting.
