# Supplementary material for: Similar Survival Rates of Territorial and Sneaker Males in a Polymorphic Damselfly: A Multi‐Year Study
Source: Ecol Evol. 2025 Dec 10;15(12):e72623. doi: 10.1002/ece3.72623 (PMC12690216; doi:10.1002/ece3.72623)
Supplement: Supplementary file 2 — Data S1: ece372623‐sup‐0002‐DataS1.zip. [file ECE3-15-e72623-s001.zip › ReadMeSummary.docx]

The meteorological data for each field season has the following variables:

Rain: Rainfall in mm

AvTemp: Average daily temperature in degrees Celsius

Sun: Hours of insolation per day

Day: Day in season (day 1 the first marking day, only observation days listed)

The mark recapture data for each field season has the following variables:

ch: Mark-recapture history (1 – first capture or resighting, 0 – no sighting)

ind: Individual

category: 1 – orange-winged male *costalis*, 2 – clear-winged male *costalis*,

3 – female *costalis*

(2007 data only): 1 – female *pruinosa*, 2 –male *pruinosa* (clear),

3 – female *costalis*, 4 – male *costalis* (orange)

size: Hindwing length in mm

asize: Abdomen length in mm (not used)

type: Morph if male (O – orange winged; C – clear winged) (not directly used)

teneral: Whether the individual was first marked as teneral (1) or not (0)
